# Woven Stainless-Steel Mesh as a Gas Separation Membrane for Alkaline Water-Splitting Electrolysis

**DOI:** 10.3390/membranes10050109

**Published:** 2020-05-23

**Authors:** William J. F. Gannon, Michael E. A. Warwick, Charles W. Dunnill

**Affiliations:** Energy Safety Research Institute, Swansea University Bay Campus, Fabian Way, Swansea SA1 8EN, UK; 920920@swansea.ac.uk (W.J.F.G.); M.E.A.Warwick@swansea.ac.uk (M.E.A.W.)

**Keywords:** membrane, water-splitting, electrolysis, alkaline, gas-separation, hydrogen production

## Abstract

A 316-grade woven stainless-steel mesh membrane was investigated as a gas-separation membrane for alkaline water-splitting electrolysis. Its resistance was measured using electrochemical impedance spectroscopy (EIS) and linear sweep voltammetry (LSV), with the conclusion that it presented approximately half the resistance of a comparable commercial alternative (Zirfon^TM^). Its gas-separation performance was analysed using gas chromatography (GC) at 140 mA cm^−2^, where it achieved 99.25% purity at the hydrogen outlet of the electrolyser. This fell to 97.5% under pumped circulation, which highlights that it is sensitive to pressure differentials. Nevertheless, this mixture is still more than a factor two inside the upper flammability limit of hydrogen in oxygen. It is hoped that such a low-cost material may bring entry-level electrolysis to many hitherto discounted applications.

## 1. Introduction

Renewable energy generation, such as solar and wind, is now the cheapest form of electricity across large parts of the world [1]. Despite that good news, it is difficult to store electrical energy without expensive, complex, and most likely imported batteries. This is where hydrogen is recognised to have the greatest scope for transformative change [2,3]. Water electrolysis is accepted as a key component of the hydrogen economy if renewable sources of energy are to be employed, and a large amount of scientific research has been dedicated to this aim over many decades [4]. Within water electrolysis, alkaline-based solutions are recognised to achieve the greatest performance in terms of capital expenditure and longevity [5], and are thus much more likely to constitute the bulk of future capacity [6,7]. In addition to the search for low-cost high-performance catalysts [8], all aspects of the water-splitting system should be investigated in the quest to drive down costs, and thus broaden the range of potential applications. To this end, an incredibly simple, low-technology gas-separation membrane would be of great utility.

**Figure 1:**
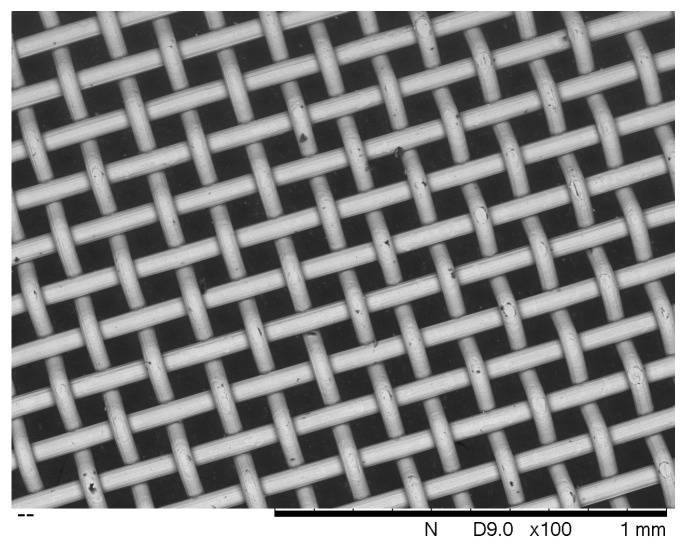
Scanning electron micrograph at ×100 magnification of 180 threads-per-inch 316-grade stainless steel woven mesh. Wire diameter: 50 μm; Membrane thickness: 100 μm–150 μm.

During experiments involving electrolyte pumped around a circuit featuring a horizontal woven stainless-steel membrane, it was observed that gas bubbles collecting underneath the mesh would not pass through. This was despite the fact that the holes in the mesh were approximately 100 μm across, and clearly visible. For reference, a scanning electron micrograph of 180 thread-per-inch woven mesh is as shown in Figure 1. It was therefore decided to investigate whether such mesh could be employed as a gas-separation membrane in its own right. From the point of view of cost this could be significant, since it has been found to be considerably cheaper than commercially available alternatives. At the time of publication, the cost of woven stainless-steel mesh (excluding sales tax) was GBP 22 per square metre (https://www.meshdirect.co.uk/woven-stainless-wire-cloth-200-mesh-0.07-mm-aperture.html viewed May 2020). As a rule, the prices of alternative membranes are not advertised, but the cost of Zirfon is known to be greater by approximately an order of magnitude. Competitor membranes such as Sustainion are newer and more expensive. Therefore, if conditions can be found under which the electrical and gas-separation capabilities of the woven membrane prove adequate, it would constitute a break-through in price and performance which could bring entry-level alkaline electrolysis within the reach of many hitherto discounted applications. This is of particular relevance to many less economically developed countries (LEDC) which have abundant sources of renewable energy, but high costs of conventional electricity, and thus offer the greatest economic case for surplus renewable energy capture by alkaline electrolysis. Despite this, the costs of commercial electrolysers are still too high.

The aim of this paper is to measure the resistance of woven stainless-steel mesh relative to that of a market-leading commercial alternative, namely Zirfon. As a result, no attempt is made to replicate the conditions inside a commercial electrolyser, which typically involve high temperature (e.g., 90 °C), high pressure (e.g., 10 bar) and high concentration (e.g., 30 wt% KOH). It shall be confirmed in future work if the relative resistance changes with concentration or temperature. In any case, room-temperature electrolysis is of greater applicability to intermittent renewable energy, where the additional electrical and system cost of heating cannot be justified. Low-pressure electrolysis is also of greater relevance to LEDC, where operating an electrolyser at atmospheric pressure significantly reduces construction costs and system complexity, and greatly increases safety.

During alkaline water-splitting electrolysis, the process of maintaining adequate separation between evolved hydrogen and oxygen gases is of paramount importance. This is not just in terms of end product purity, but also in terms of system safety. It is an accepted industry standard that commercial hydrogen intended for use with fuel-cell vehicles, such as cars or trains, must achieve ‘five nines’ levels of purity, or 99.999% [9]. However, this high level of purity stems primarily from the most common method of hydrogen production, that being the steam-reforming of hydrocarbons such as natural gas or methanol. In this case the main contaminant is CO, which is known to ‘poison’ fuel-cells by preferentially adsorbing to platinum, thereby reducing the catalyst area available to hydrogen, and greatly reducing efficiency. In the case of hydrogen-powered vehicles, this limits the engine power available, which could be a serious safety issue, or make the difference between climbing a hill or not [10].

Deliberate or accidental contamination of hydrogen with small quantities of oxygen is in fact desirable from an end-use point of view, particularly if that end use is a fuel-cell. The introduction of 2 to 5% oxygen into the hydrogen supply (specifically to the anode) is a recognised mitigation scheme known as ‘O_2_ bleeding’, that permits the fuel-cell to cope with CO concentrations up to 500 ppm. It is able to do this because it substantially increases the rate at which adsorbed CO is oxidised to CO_2_ [10]. In addition, the small amounts of waste water generated can assist in maintaining an adequate level of hydration of fuel-cell membranes, such as Nafion^TM^. Hydration is critical for all aspects of the performance of the membrane, not least its primary purpose of conducting protons [11].

In both conventional and zero-gap alkaline electrolysis, gas-separation is normally achieved by the use of a dedicated membrane. One well-known commercial product is Zirfon^TM^, which is constructed of a relatively soft, spongey material (a mixture of polymer and zirconium oxide) surrounding a hard woven mesh (made of polyphenylene sulphide) [12]. The membrane works via a combination of its chemical stability and high wettable area. Zirconium oxide is recognised for being chemically unreactive, and is able to withstand extended exposure to concentrated alkali solutions [13]. Its resistance in 30 wt% KOH has been measured as 0.3 Ω cm^2^ [7].

Fumasep FAS-50 is an anion exchange membrane (AEM) with a thickness of 50 μm. It therefore operates by the direct conduction of OH^−^ ions along polymer chains, in a similar way that Nafion conducts H^+^ ions. It has a list price of USD 783 per square metre (source: https://www.fuelcellstore.com/fumasep-fas-50 viewed May 2020), and exhibited an impedance of 0.3783 Ω cm${}^{2}$ in 1 M KOH at 60 °C. However, the voltage required to sustain 1 A cm${}^{-2}$ was observed to increase by 200 μV h^−1^ to 400 μV h^−1^, and the membrane failed completely after 200 h, even though the membrane should have been stable at pH 14 [14].

Sustainion is an AEM with a thickness of 50 μm, comprised of imidazolium functionalized styrene polymer. It has a list price of USD 6304 per square metre (source: https://www.fuelcellstore.com/sustainion-x37-50-grade-60-membrane viewed May 2020), and exhibited the lowest reported resistance of just 0.045 Ω cm${}^{2}$ [14]. However, the strength of the electrolyte is limited to 1 M KOH “due to the lack of chemical stability of the materials in a very strongly basic environment”. The commercial use of any samples purchased is also prohibited by an End User Agreement. The membrane exhibited a gas-crossover rate 20 times higher than Zirfon in 30 wt% KOH at all current densities from 100 mA cm${}^{-2}$ to 2000 mA cm${}^{-2}$ [15]. It has also proved mechanically fragile, and extremely easy to perforate during electrolyser assembly and disassembly.

Other membranes include Neosepta, AMI 7001 and Celazole PBI, but all exhibited higher resistances than the above three materials [14]. It is also possible to maintain gas-separation without a membrane, for example by using a rotating electrolyser [16], or divergent electrolyte flow [17], and these are certainly concepts worthy of consideration. It is also possible to mix the evolved hydrogen and oxygen gases, as a stoichiometric mixture known as ‘HHO’, thereby avoiding the need for a membrane, so long as the gas is for immediate use with zero storage [18].

## 2. Results

**Figure 2:**
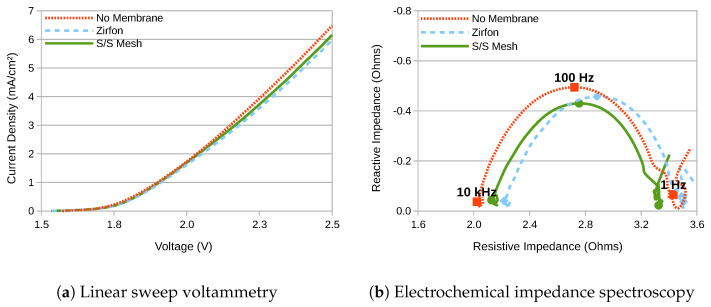
LSV and EIS characteristics obtained with two gas-separation membranes, plus with no membrane for comparison.

The LSV and EIS results for the three different membrane options are as shown in Figure 2. At first glance, it appears that the choice of membrane makes little difference, but this is misleading. This is because the test cell was designed for experimentation, not for efficiency, and the solution resistance of the electrolyte constitutes a much higher proportion of the total than would normally be the case. The results confirm that performance is highest with no membrane at all, as is to be expected since this configuration does not separate the product gases, and is included solely as a baseline for performance comparison. Between the two membranes, the woven stainless-steel demonstrates performance that is approximately halfway between that of Zirfon and no membrane.

More quantitatively, the series electrical resistance ($R_{S}$) between the electrodes can be calculated from the EIS results at high frequency, its value being given by the point of intercept closest to the origin between the semi-circle and the x-axis [14]. Since the semi-circles did not quite intercept the x-axis, this has been taken to be the minimum value of the real component of the impedance, as presented in Table 1.

The 36 cm${}^{2}$ stainless-steel mesh therefore exhibited a resistance that was 90 mΩ lower than the same area of Zirfon membrane, which equates to a reduction of 3.2 Ω cm${}^{2}$ or 44%. The absolute value measured for the area resistance of the Zirfon membrane at 7.2 Ω cm${}^{2}$ demands further attention, since the published value for this by Vermieren et al. is more than 30 times lower at 0.2 Ω cm${}^{2}$. However, this was measured using pre-production samples in a much stronger electrolyte (30 wt% KOH) and at a slightly higher temperature ( 30 °C) [12,19]. A slightly higher figure of 0.3 Ω cm${}^{2}$ was produced by Rodríguez et al., who also used 30 wt% KOH. However, another measurement by R. Phillips placed its area resistance at 1.2 Ω cm${}^{2}$ in 1M NaOH at 20 °C as part of a zero-gap electrolyser [15,20,21].

If it is assumed that 0.5M NaOH has approximately double the electrical resistance of 1M NaOH, this still means that the area resistance measured for Zirfon is three times higher than it should be. However, the measurements by Vermeiren and Rodríguez et al. were performed by pressing electrodes into close contact with each side of the membrane, whereas those by R. Phillips were performed in a zero-gap electrolyser with copious electrolytic circulation. It is known from experiments with ion-exchange membranes that significant additional resistances occur due to ionic transport through diffusion boundary layers and electrical double-layers that occur each side of the membrane [22]. Therefore, in relatively low concentration uncirculated electrolytes where the resistance due to diffusion is greater, a figure of 7.2 Ω cm${}^{2}$ is a possibility.

**Figure 3:**
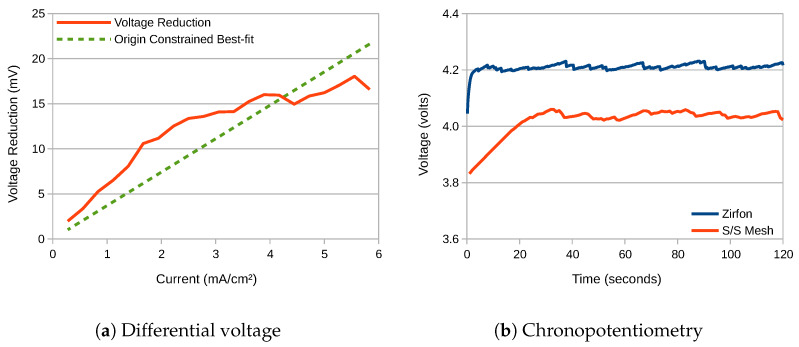
(**a**) Voltage improvement versus current observed for a 2-electrode electrolyser featuring a stainless-steel mesh membrane relative to Zirfon. (**b**) Voltage variation versus time for the same two membranes. Current density: 25 mA cm^−2^

In any case, the EIS-based measurement of membrane resistance can be cross-checked by analysing voltage differentials at equal currents in Figure 2a. Since the kinetic overpotentials at the anode ($\eta_{a}$) and the cathode ($\eta_{c}$) are a function of current, and the thermodynamic water-splitting potential ($V_{th}$) is only a function of temperature, it is possible to state that:

(1) $$\begin{array}{cc} V_{1}-\eta_{a}-\eta_{c}-V_{th} & =I(R_{S}+R_{M1})\\ V_{2}-\eta_{a}-\eta_{c}-V_{th} & =I(R_{S}+R_{M2})\\ \Rightarrow R_{M2}-R_{M1} & =\frac{V_{2}-V_{1}}{I} \end{array}$$

where $R_{s}$ is the non-varying solution resistance of the electrolyte, and $V_{1}$ and $V_{2}$ are the total voltages measured at current *I* for the membranes with resistance $R_{M1}$ and $R_{M2}$ respectively. A plot of $V_{2}-V_{1}$ against current derived from Figure 2a appears as presented in Figure 3a. The figure includes an origin-constrained line of best-fit, the slope of which is therefore equal to $R_{M2}-R_{M1}$. This is measured as 3.7 Ω cm${}^{2}$, which compares well with the figure of 3.2 Ω cm${}^{2}$ generated from EIS.

It is possible to state how much of a voltage difference this would make at different current densities. Since a current density of 500 mA cm${}^{-2}$ would equate to 18 A, this would result in a voltage reduction of:

(2) 18A×90mΩ=1.62 V

This figure serves to highlight how high the resistance of 0.5 M NaOH is. Since not just the resistance of the electrolyte is increased, but also that of the membrane and any diffusion boundary layers, increasing the conductivity of the electrolyte is critical. This is highlighted in the chronopotentiometry waveforms presented in Figure 3b, which were conducted at a current density of 25 mA cm${}^{-2}$. The woven mesh membrane produced an average terminal voltage just over 150 mV lower than Zirfon. The waveforms present the characteristic ‘saw-tooth’ profile that is the result of the build-up and sudden release of bubbles on the electrode surfaces, which is not attributable to the membrane.

### 2.1. Gas Chromatography

**Figure 4:**
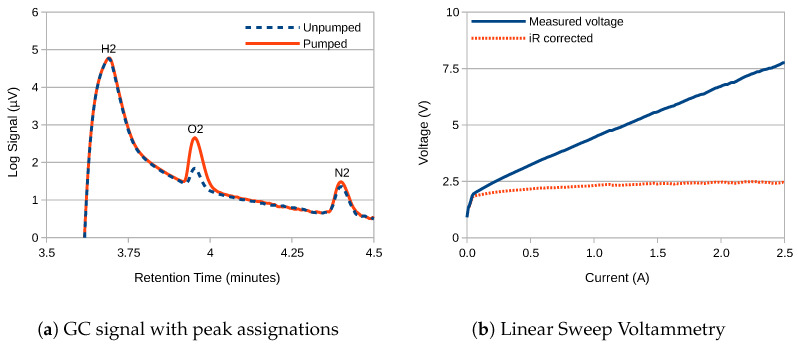
(**a**) Gas chromatography (GC) measurements of gas purity of the hydrogen outlet of the electrolyser with and without pumped circulation of the electrolyte. Gas-separation membrane: 180 threads-per-inch 316-stainless steel woven mesh; electrolyser current: 5 A; electrolyte: 3 M NaOH. (**b**) Linear sweep potentiometry (LSP) between 0A and 2.5A to check for evidence of bipolar-electrode behaviour. No such behaviour is observed.

The gas chromatography results for the hydrogen outlet of the electrolyser are as presented in Figure 4a. The gas-separation membrane consisted of 180 threads-per-inch 316-grade stainless-steel woven mesh. The electrolyser current was 5 A, which equated to 140 mA cm${}^{-2}$. Separate measurements were taken with and without pumped circulation of the electrolyte. Peak-fitting analysis within the GC system software against a recent calibration produced the gas purity figures presented in Table 2. The figures have been corrected for contamination by atmospheric air in-line with the method outlined in Section 4.4.

### 2.2. Bipolar Operation

Since the woven stainless-steel membrane is itself conductive, the possibility exists that instead of behaving like a porous membrane, at some current density it will begin to behave like a bipolar-electrode. In this situation, hydrogen and oxygen gas would be evolved on opposite sides of the separator, and the electrolytic cell would start to behave like a two-cell bipolar electrolyser. As such, the rate of increase of the total voltage with current density would be expected to increase, since extra voltage would be needed to account for the extra water-splitting reactions. In addition, the purity of the gases at the outlets would decrease, since a mixture of both gases would be evolved in each half of the electrolyser.

The point at which ‘membrane behaviour’ gives way to ‘bipolar-electrode behaviour’ will depend on the magnitude of the charge transfer resistance of the water-splitting reactions versus the electrolyte resistance through the membrane. This in turn will depend on the size of the holes in the mesh. As proof, it is simple to envisage a thought experiment whereby a solid metal sheet (which will by definition behave like a bipolar-electrode) is altered gradually through the inclusion of holes first into a perforated mesh, then into a woven mesh, then into no membrane at all. At some point, as the hole/metal ratio increases, the behaviour will change from bipolar-electrode to porous membrane.

To investigate this, the cell was swept from 0 A to 2.5 A, with the results as presented in Figure 4b. This represented the maximum current available from this particular model of potentiostat. Since the series resistance of the cell is known to be 2.13 Ω (see Table 1) it is possible to calculate the iR-corrected voltage, which is displayed as the dotted orange line. This shows that the proportion of the voltage used for water-splitting does not exceed 2.5 V, which is consistent with a single water-splitting reaction and rules out any bipolar-electrode behaviour. However, given the size of the membrane, this equates to a current density of just 70 mA cm${}^{-2}$, which is well below that typically seen in a commercial electrolyser. To take the current density higher would require either a higher capacity potentiostat, or a redesign of the electrochemical cell, both of which are left for future endeavour.

## 3. Discussion

The gas purity calculations presented in Table 2 show that pumping of the electrolyte increases the concentration of O_2_ in the H_2_ outlet by more than a factor of three. This is to be expected, since the woven membrane is much more porous than Zirfon, and will therefore be much more sensitive to pressure differentials across it. No attempt was made to equalise the pressures of the two pumps, since it was believed this might create an unrealistic expectation of the performance of the membrane. However, it is quite possible to design electrolysers such that pressure differentials across the membrane are kept to an absolute minimum, even with pumped circulation.

The upper flammability limit (UFL) for H_2_ mixed with pure O_2_ is 94% [23] (i.e., 6% O_2_ in H_2_) therefore the figure of 2.52% under pumped circulation is still more than a factor of two within the limit. It is, however, more conventional to measure the gas-purity of the oxygen outlet [24,25], since the lower flammability limit (LFL) for H_2_ in O_2_ appears a more severe constraint at just 4% [23]. However, this can be misleading, since the gas produced at the oxygen outlet is not normally stored, but instead immediately vented to the atmosphere [26]. Nevertheless, there are some applications where oxygen storage is beneficial, for example to increase the maximum power available from a fuel-cell [27], and for these some form of outlet gas purification such as a heated platinum wire could be considered [28].

In conclusion, it is possible to report that the woven stainless-steel mesh membrane:

- Presents approximately half the electrical resistance of commercial Zirfon^TM^ gas-separation membrane. This could result in a significant efficiency saving in most applications, where the resistance of the electrolyte constitutes a smaller proportional of the total,
- Maintained gas-separation such that at the H_2_ outlet of the electrolyser only 0.75% O_2_ was observed (with uncirculated electrolyte, at a current density of 140 mA cm${}^{-2}$),
- Produced 2.5% O_2_ at the H_2_ outlet with pumped circulation, most likely due to an uncompensated pressure differential across the membrane. This is still more than a factor of two below the upper flammability limit (UFL) of hydrogen in oxygen,
- Has been found to be considerably cheaper than commercially available alternative membrane materials

It is therefore possible that woven stainless-steel mesh membrane could prove to be a cost-effective gas-separation membrane in some applications. It is also possible that its properties could be further enhanced using suitable structural modifications or coatings.

## 4. Materials and Methods

All membranes and electrodes were mounted in a 3-electrode cell constructed in a laminar fashion from laser-cut acrylic [29,30]. The exposed area of all membranes was 6cm×6 cm, and the distance between anode and cathode was 36 mm. The distance between electrodes was large to increase repeatability, both by reducing the effect of cell assembly variation, and by reducing void fraction under gas evolution. An extra silicone gasket was inserted when no membrane was present to maintain inter-electrode spacing. The stainless-steel membrane consisted of woven mesh with 180 threads per inch and was obtained from a commercial supplier. The Zirfon^TM^ membrane was obtained from a commercial supplier, and had a thickness of 0.9 mm. Other membrane materials are available, but were not part of this study [14].

The exposed area was 3cm×3 cm for the working electrode (WE, cathode), which had been coated with Raney Nickel version 1 in accordance with previous instructions [31]. The exposed area was 6cm×6 cm for the 316-grade stainless-steel counter electrode (CE, anode). The reference electrode (RE) was a commercial Ag/AgCl design, which was routinely corrected against a Standard Calomel Electrode (SCE). The cell evolves hydrogen and oxygen gas bubbles, which are kept apart by the membrane, thereby ensuring gas purity at each gas outlet. For experiments on the potentiostat the electrolyte was 0.5 M NaOH with no pumped circulation, at normal laboratory temperature, which was 21 ± 1 °C. For experiments on the GC the electrolyte was 3 M NaOH with optional pumped circulation, at normal laboratory temperature plus some self-heating.

All electrochemical experiments were performed on an Ivium n-Stat potentiostat. All EIS results were analysed within the IviumSoft software package. **Safety:** The electrolysis of water evolves small amounts of hydrogen and oxygen gas, which constitute a risk of explosion. The experiments were therefore performed with fume extraction, so as to prevent the build-up of gases and to achieve isolation from possible sources of ignition. Strong electrolyte up to 3 M NaOH was used, which is dangerous to skin and eyes, therefore normal laboratory eye protection and gloves were employed.

### 4.1. Linear Sweep Voltammetry

LSV was performed using 2-electrodes (CE and WE) between 1.4 V and 2.5 V at 20 mV $s^{-1}$. A temperature probe was used to measure the temperature of the electrode inside the electrochemical cell, which was higher than ambient temperature due to self-heating, but within 2 °C.

### 4.2. Electrochemical Impedance Spectroscopy

EIS was performed between 0.1 Hz and 10 kHz, starting at low frequency, at a bias voltage of 2 V to ensure water-splitting reactions were present. Method: impedance; technique: constant voltage; signal magnitude: 10 mV. The electrolytic cell was pretreated for 60 s at 2 V to reduce initial transient currents.

### 4.3. Gas Chromatography

**Figure 5:**
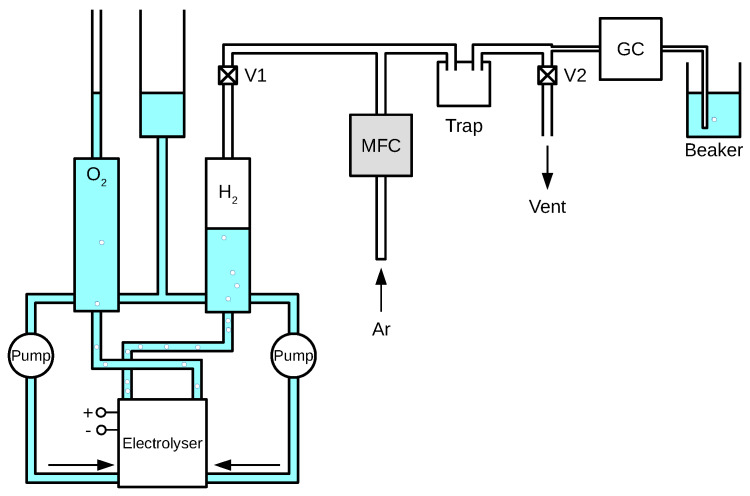
Experimental set-up for electrolyser gas-chromatography.

GC experiments were performed on an Agilent Technologies 7820A GC System with OpenLAB CDS ChemStation Edition software. The experimental set-up was as shown in Figure 5. The total amount of electrolyte was under 500 ml. The rate of argon flow through the Mass Flow Controller (MFC) was configured at 10 ml $\min^{-1}$, whereas the rate of gas flow through the GC (prior to sampling) was much lower at ∼1 ml $\min^{-1}$. Therefore, with valve V1 open and V2 shut, argon would back-up into the H_2_ collection tank. This was subsequently used to flush the line by opening valve V2, after which the MFC would be switched off and a new sample of electrolyser gas collected. Whilst this sample was flushing out the Ar, and after leaving enough time for the sample gas to travel through the last section of tube, the start button would be pressed, and the GC would begin its automated analysis cycle. The amount of Ar or H_2_ gas collected was about 100 ml, which was chosen since it was approximately double the total volume of the tubes and trap between V1 and the GC.

### 4.4. Correction for Contamination by Atmospheric Air

Due to saturation of the detector within the GC, and since the concentration of H_2_ was far higher than that used during calibration, it was known that any concentrations reported for H_2_ would be incorrect, and were thus discounted. Instead, the concentration of N_2_ was used to correct the concentration of O_2_, on the assumption that all of the N_2_ present had arisen due to contamination by atmospheric air, which was assumed to be 78% N_2_ and 21% O_2_. The corrected concentration of O_2_ was then subtracted from 100% to produce the concentration of H_2_.

## Figures and Tables

**Table 1:** Series electrical resistance between the electrodes derived from the EIS results presented in Figure 2b. The membrane area resistance $R_{M}$ is derived from the value of $\Delta R_{S}$. The woven stainless-steel membrane therefore exhibited just over half the electrical resistance of Zirfon.

Membrane	$R_{S}$	$\Delta R_{S}$	$R_{M}$
No Membrane	2.02 Ω	–	–
S/S Mesh	2.13 Ω	+110 mΩ	4.0 Ω c m ${}^{2}$
Zirfon	2.22 Ω	+200 mΩ	7.2 Ω c m ${}^{2}$

**Table 2:** Gas purity calculations based on the data presented in Figure 4a.

Name	Peak Area	Response Factor	Amount (%)	Corrected (%)
**H_2_** (not pumped)	5454	0.00852	46.47	99.25
**O_2_**	12.9	0.06162	0.80	0.75
**N_2_**	2.17	0.08184	0.18	0.00
**H_2_** (pumped)	5734	0.00852	48.85	97.48
**O_2_**	41.9	0.06162	2.58	2.52
**N_2_**	2.89	0.08184	0.24	0.00

## Abbreviations

The following abbreviations are used in this manuscript:

EIS	Electrochemical Impedance Spectroscopy
LSV	Linear Sweep Voltammetry
GC	Gas Chromatography
LFL	Lower Flammability Limit
UFL	Upper Flammability Limit
WE	Working Electrode
CE	Counter Electrode
RE	Reference Electrode
SCE	Standard Calomel Electrode
LEDC	Less Economically Developed Countries
